# The Prognostic Value of Pyrosequencing-Detected MGMT Promoter Hypermethylation in Newly Diagnosed Patients with Glioblastoma

**DOI:** 10.1155/2015/604719

**Published:** 2015-08-11

**Authors:** Veronica Villani, Beatrice Casini, Andrea Pace, Luca Prosperini, Carmine M. Carapella, Antonello Vidiri, Alessandra Fabi, Mariantonia Carosi

**Affiliations:** ^1^Neuro-Oncology Unit, “Regina Elena” National Cancer Institute, Via Elio Chianesi 53, 00144 Rome, Italy; ^2^Division of Pathology, “Regina Elena” National Cancer Institute, Via Elio Chianesi 53, 00144 Rome, Italy; ^3^Department of Neurology and Psychiatry, Sapienza University, Viale dell'Università 30, 00185 Rome, Italy; ^4^Division of Neurosurgery, “Regina Elena” National Cancer Institute, Via Elio Chianesi 53, 00144 Rome, Italy; ^5^Service of Neuroradiology, “Regina Elena” National Cancer Institute, Via Elio Chianesi 53, 00144 Rome, Italy; ^6^Division of Medical Oncology, “Regina Elena” National Cancer Institute, Via Elio Chianesi 53, 00144 Rome, Italy

## Abstract

O6-methylguanine-DNA-methyltransferase (MGMT) has emerged as a relevant predictor of therapeutic response and good prognosis in patients with glioblastoma (GBM). Transcriptionally active MGMT rapidly removes the alkyl adducts, preventing the formation of cross-links and thereby causing resistance to alkylating drugs. Studies with pyrosequencing (PSQ) showed that this technique has a higher reproducibility and sensitivity than other techniques. However, the definition of a prognostically relevant threshold for the percentage of MGMT methylation remains one of the most critical issues in the use of PSQ analysis. The aim of this study was to define the cut-off value correlated with good favourable prognostic outcomes. We retrospectively analyzed 51 patients (33 males, 18 females) with GBM who underwent surgery or biopsy. The Receiver Operating Characteristics analysis showed that the best possible criteria for PSQ-detected percentage of MGMT methylation that predicted progression-free survival (PFS) and overall survival (OS) were 19% and 13%, respectively. Patients with ≤19% of PSQ-detected MGMT had a shorter PFS (HR: 0.24, *p* < 0.01); those ones with ≤13% had a shorter OS (HR: 0.33, *p* < 0.05). Our study reinforces the importance of MGMT in the management of GBM patients, but future studies with larger sample sizes are warranted to confirm our findings.

## 1. Introduction

Glioblastoma multiforme (GBM) is the most frequent primary brain tumour in adults and is associated with poor prognosis [1]. The standard of care for patients with GBM currently involves surgical resection and temozolomide chemotherapy with concomitant radiotherapy, followed by cycles of adjuvant temozolomide [1]. Although some clinical trials have recently demonstrated that the standard treatment improves overall survival, only one-third of GBM patients seem to benefit from these therapies. Alkylating chemotherapeutic agents, such as temozolomide, induce cell death by forming cross-links between adjacent DNA strands through alkylation of the O6 position of guanine [2]. Transcriptionally active O6-methylguanine-DNA-methyltransferase (MGMT) rapidly removes the alkyl adducts, preventing the formation of cross-links, thereby causing resistance to alkylating drugs [3].

The loss of MGMT protein expression caused by hypermethylation of the MGMT promoter reduces the DNA repair activity of glioma cells, overcoming their resistance to alkylating agents [2, 4–6]. Therefore, methylation of MGMT has become a clinically relevant predictor of response to treatment in glioma patients [7–10].

MGMT promoter hypermethylation is associated with longer progression-free and overall survival in patients who receive alkylating chemotherapy in association with radiotherapy [10]. MGMT methylation status is currently incorporated into a more refined classification system and applied in the clinical decision-making process, but there is no evidence yet about what is the most accurate diagnostic tool to estimate MGMT promoter hypermethylation. Most studies used methylation-specific polymerase chain reaction (MS-PCR) [8, 11–13]; however, one of the major drawbacks of this technique is its operator-dependent nature, mainly due to the sample reading subjectivity and lack of automation. Studies that compared various techniques for the assessment of MGMT methylation status showed that pyrosequencing (PSQ) shows a better prediction of survival, in addition to higher reproducibility and sensitivity with respect to other techniques [12, 14–16]. However, only few studies have investigated which is the most accurate cut-off value that could represent methylated or unmethylated status. Consequently, the definition of a prognostically relevant threshold for the percentage of MGMT methylation remains one of the most critical issues in the use of PSQ analysis [17–21]. In clinical practice methylation is very important because it is considered a strong predictor of response to chemotherapy with alkylating agents and may help to drive personalized treatment strategies.

Starting from these assumptions, in this study we aimed to define the best cut-off value for PSQ-detected MGMT promoter hypermethylation which correlated with the most favourable prognostic outcomes.

## 2. Materials and Methods

### 2.1. Patients

Data of patients affected by newly diagnosed primary GBM who underwent surgery or biopsy and followed at the Neuro-Oncology Unit of Regina Elena National Cancer Institute were retrospectively analyzed. Tissue samples were matched with a comprehensive set of clinical data collected in the database of Neuro-Oncology Unit for each patient. The database includes demographic, clinical, and molecular data, as well as data on response to treatments and outcomes, including progression-free survival (PFS) and overall survival (OS), which was considered the main outcomes of this study. PFS was defined as the time elapsed from the first day of treatment and the date on which disease “progresses” or the date on which the patient dies. OS was defined as the time elapsed from first day of treatment and the date of death.

### 2.2. Tissue Analysis

Tissue samples were analyzed by means of PSQ for methylation status of MGMT assessment according to standardized procedure.

Genomic DNA was isolated from 4-5 paraffin sections of glioblastoma tissue. At least 1 slice was stained with hematoxylin and eosin to control the percentage of tumor cells. Tumor samples with at least 70% of tumor cells were considered, and DNA extraction was performed with QIAamp DNA FFPE tissue (Quiagen). The extracted DNA was subjected to polymerase chain reaction (PCR) amplification with a forward primer and a biotinylated reverse primer using the “MGMT PLUS” kit (Diatech pharmacogenetics) and the “Rotor-Gene TM 6000” instrument. The PCR condition for MGMT gene was 95°C for 5 minutes, 45 cycles of 95°C for 30 seconds, 53°C for 30 seconds, and 72°C for 20 seconds, 72°C for 5 minutes, and then Green signal acquisition at 60°C for 20 seconds. During amplification, the uracil product by modification of cytosine unmethylated is converted in thymine while 5-methylcytosine remains as cytosine; therefore we can distinguish, in the sequence, methylated from unmethylated cytosine. We performed PSQ methylation assay to evaluate 10 CpG sites in the following regions: chr 10: 131,265,507–131,265,556 using sequencing primer of MGMT Kit Diatech (Pharmocogenetics, Jesi, Ancona, Italy). PSQ analysis was performed with PyroMarker CpG software 1.0.11 (Qiagen). The software gives a mean methylation value for each 10 CpG site and the total mean of all 10 CpG sites.

### 2.3. Statistical Analysis

Descriptive statistics were used to summarize pertinent study information. Categorical variables were reported as frequencies and percentage values, while continuous variables were reported as mean values and their relative standard deviation (SD), or median (range), as appropriate.

Since PSQ provided a percentage of MGMT methylation, the optimal cut-off value identifying methylated or unmethylated status was estimated by using the Receiver Operating Characteristics (ROC) curve analysis. Across various cut-off points, Youden's index maximised the differences between real-positive and false-positive subjects; thus, the optimal cut-off values predicting overall survival and progression-free survival were estimated.

To investigate the prognostic relevance of the PSQ-detected MGMT promoter hypermethylation, univariate and multivariable Cox proportional hazards models were carried out by considering separately the overall survival and the progression-free survival as dependent variables. Outcome measures were evaluated after 1 year of follow-up. Gender, age at diagnosis, surgery (extent of removal/biopsy), radiotherapy (yes/no), and chemotherapy (yes/no) were inserted into the models as covariates and percentage of MGMT methylation was inserted as independent variable. Additional univariate and multivariable Cox proportional hazards models were built by replacing the percentage of MGMT methylation with a dichotomous variable derived from the afore described ROC analyses.

Lastly, since MGMT promoter hypermethylation is known to impact the response to treatment [17], Cox regression analyses were repeated only in the subsample who were treated with Stupp regimen [1].

All *p* values less than 0.05 (two-sided) were considered as significant. Data were analysed with the Statistical Package for Social Sciences, version 21.0 (IBM SPSS, Chicago, IL, USA).

## 3. Results

### 3.1. Participants

In total, 51 patients (33 men, 18 women) diagnosed as affected by GBM from June 2013 to March 2015 were included in this study.  Table 1 shows the characteristics of the study sample. The mean (SD) age of the patients was 61.7 (12.9) years. There were no significant differences between women and men in terms of baseline demographic and clinical characteristics. The mean and median percentage of MGMT methylation, as detected by PSQ, were 21.5% and 16% (ranging from 2 to 85). No significant relationships were observed between gender, age at diagnosis, time from disease onset to diagnosis and percentage of MGMT methylation.

### 3.2. Follow-Up Data

The median follow-up time was 12 months (ranging from 3 to 27 months).

PFS: thirty-four (64%) patients experienced disease progression after a mean time of 12.1 (5.6) months from the diagnosis (ranging from 4 to 18).

OS: twenty-four (47%) patients died after a mean follow-up time of 12.6 (7.2) months from the diagnosis (ranging from 3 to 27), while the mean follow-up time from the diagnosis to last visit was 11.3 (6.5) months for the 27 survivors (53%) (ranging from 3 to 26) (*p* = 0.5).

### 3.3. Receiver Operating Characteristics Analyses

The main findings of the ROC analyses are summarized in Table 2. The best possible criteria for PSQ-detected percentage of MGMT methylation that predicted PFS and OS were 19% and 13%, respectively. Adopting a cut-off value of 19% of MGMT methylation ensured a sensitivity of 73% and specificity of 77% in predicting progression-free survival (*p* = 0.0054). Adopting a cut-off value of 13% of MGMT methylation ensured a sensitivity of 58% and specificity of 70% in predicting overall survival (*p* = 0.057).

### 3.4. Time-to-Event Analyses

**Figure 1:**
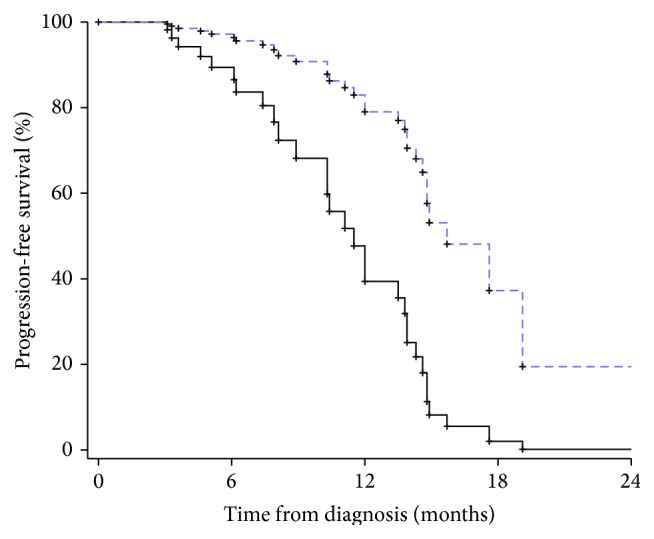
Kaplan-Meier curve showing the time to progression according to the percentage of MGMT methylation (<19%: continue line; ≥19% dotted line).

**Figure 2:**
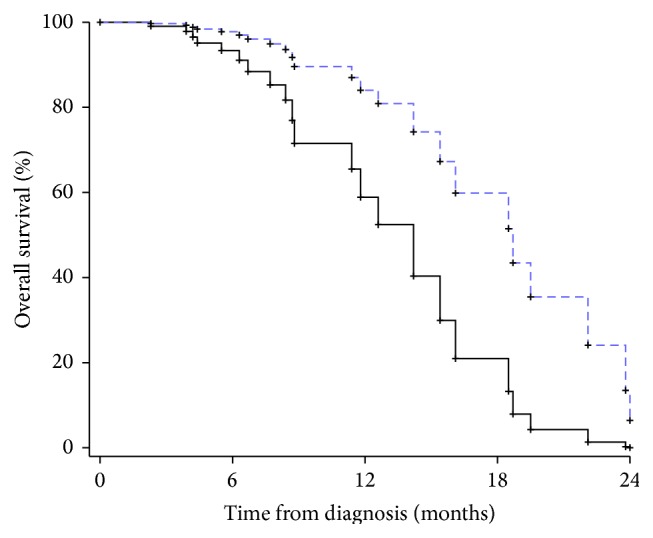
Kaplan-Meier curve showing time to death according to the percentage of MGMT methylation (<13%: continue line; ≥13% dotted line).

Kaplan-Meier curves show that patients who had less than 19% and 13% of MGMT methylation experienced worse PFS and OS, respectively (both *p* values < 0.05 by the Log-Rank test; see also Figures 1 and 2).

In the final Cox models, the variables which resulted in predicting the PFS and OS were those shown in Tables 3 and 4, respectively.

The risk of disease progression was reduced by 3% for each unit of increase in percentage of MGMT methylation (HR: 0.97, 95% CIs 0.94–0.99, *p* < 0.01). Consistently, patients who presented more than 19% MGMT methylation had a 76%-decreased risk of disease progression (HR: 0.24, 95% CIs 0.10–0.64, *p* < 0.01).

While the percentage of MGMT methylation did not contribute to fitting the multivariable analysis predicting OS (and thereby was excluded from the model), those patients who presented a MGMT methylation above 13% had a 67%-reduced risk of death (HR: 0.33, 95% CIs 0.12–0.92, *p* < 0.05).

### 3.5. Additional Analysis on Patients Treated with Stupp Regimen

A total of 32 patients (63% of the whole study sample) were treated with Stupp regimen and were followed up to a median time of 15 months (ranging from 4 to 27 months). Despite the reduced size of this subsample, more than 19% of MGMT methylation was still predictive of the risk of disease progression (HR: 0.16, 95% CIs 0.03–0.73, *p* < 0.05). By contrast, the cut-off value of 13% of MGMT methylation did not predict OS in the subgroup of patients treated with Stupp regimen (HR: 0.35, 95% CIs 0.10–1.27, *p* = 0.1), probably due to the small sample size.

## 4. Discussion

MGMT methylation status is considered an important marker for the prognosis and therapeutic response of patients with newly diagnosed GBM who are treated with standard care [1]. However, there are still some open questions, mainly concerning what is the best technique for the assessment of methylation and what is the optimal threshold indicating methylated or unmethylated status. Recent studies have reported that the best predictive value was obtained by PSQ compared to other techniques [12], but cut-off values for the percentage of methylation that define the methylation status remain one of the most critical issues. PSQ allows highly reproducible quantitative evaluation of methylation at discrete CpG sites therapy providing more information on promoter methylation patterns. Locus-specific hypermethylation, mostly at the CpG island (CGI) promoters, is frequent in patients with GBM. CGIs are regions of about 500 bp to 1 kb in which CpG nucleotides are approximately five times more abundant compared to the rest of the genome. MGMT encodes a DNA repair protein that removes alkyl adducts at the O6 position of guanine. MGMT expression protects normal cells from carcinogens; however, it can also protect cancer cells from chemotherapeutic alkylating agents [18]. Therefore, MGMT status is a strong predictor of response to treatment with temozolomide and it is determined in most ongoing clinical trials using this drug [1, 7, 8].

Most studies reporting a link between MGMT status and survival in patients with GBM have used PSQ [17–21]. Findings from the present study confirm that the percentages of methylation estimated by PSQ are highly correlated with prognostic outcome in these patients. In the present study, the best possible criteria for PSQ-detected percentage of MGMT methylation that predicted PFS and OS were 19% and 13%, respectively. These data slightly deviate from literature data that reported lower cut-off values, ranging from 8% to 10% [17, 20, 22]. Dunn et al. [17] considered methylated those patients who had ≥9% average methylation and unmethylated those ones who had an average methylation <9% in all samples. Mikeska et al. [20] reported that unmethylated tumor samples and control samples showed ratios of <10% at all positions with a small SD and suggested a score to separate unmethylated and methylated cases by employing the percentage values of four specified CpGs. Wiewrodt et al. [22] showed that patients expressing ≤30 fmolmg-1 MGMT protein in the pretreatment tumor volume had a significantly better response to alkylating therapy than those with MGMT protein above this level.

However, consistently with literature data, we observed that PSQ had good sensitivity and specificity (73% and 77%, resp.) in predicting PFS (*p* = 0.0054), while its sensitivity decreases to 58%, and specificity remains good (70%), in predicting OS (*p* = 0.057).

In our study, the risk of disease progression was reduced by 3% for each unit increase in percentage of MGMT methylation (HR: 0.97, 95% CIs 0.94–0.99, *p* < 0.01). Consistently, patients who presented more than 19% MGMT methylation had a 76%-decreased risk of disease progression (HR: 0.24, 95% CIs 0.10–0.64, *p* < 0.01). Also patients who presented a MGMT methylation above 13% had a 67%-reduced risk of death (HR: 0.33, 95% CIs 0.12–0.92, *p* < 0.05). The additional analysis only on patients treated with Stupp regimen [1] strengthens our findings and reinforces the role of MGMT methylation in predicting the response to treatment, at least in terms of PFS [17].

Taken together, these results support the hypothesis that MGMT methylation is a relevant prognostic marker since it impacts on disease progression and survival. For this reason, we strongly recommend molecular assay for the assessment of MGMT status in the management of all patients affected by newly diagnosed GBM. The main limit of the present study is the small sample size. For this reason, future studies on larger population are warranted to confirm the cut-off values we have found as the best possible predictive criteria of good prognosis.

## Figures and Tables

**Table 1:** Demographic and clinical features of the study sample (*n* = 51).

*Baseline characteristics *	
Gender, *n* (%)	
Women	18 (35%)
Men	33 (65%)
Age, years	
Mean (SD)	61.7 (12.9)
Median (range)	62 (25–84)
MGMT methylation, %	
Mean (SD)	21.5 (19.1)
Median (range)	16 (2–85)
*Treatment history *	
Surgery, *n* (%)	
Done	46 (90%)
Not done	5 (10%)
Radiotherapy, *n* (%)	
Done	39 (76%)
Not done	12 (24%)
Chemotherapy, *n* (%)	
Done	41 (80%)
Not done	10 (20%)
*Main outcomes *	
Overall survival, *n* (%)	
Dead	24 (47%)
Alive	27 (53%)
Progression-free survival	
Progressed	34 (67%)
Not progressed	17 (23%)
Follow-up time, months^*∗*^	
Mean (SD)	11.9 (6.8)
Median (range)	12 (3–27)

^*∗*^Follow-up time refers to time from disease diagnosis and death or last visit.

**Table 2:** Receiver Operating Characteristic (ROC) analyses showing the best cut-off values of MGMT predicting overall survival (left) and progression-free survival (right) in the study sample (*n* = 51).

	Progression-free survival	Overall survival
AUC (95% CIs)	0.71 (0.57–0.83)	0.65 (0.50–0.78)
Criterion (cut-off)	≥19%	≥13%
Sensitivity	73%	58%
Specificity	77%	70%
*p* value	***0.0054***	0.057

AUC: area under the curve; MGMT: O6-methylguanine-DNA-methyltransferase.

**Table 3:** Cox proportional hazard models predicting progression-free survival (dependent variable).

	Univariate analysis			Multivariable analysis		
	HR	95% CIs	*p* value	HR	95% CIs	*p* value
Gender (men versus women)	1.20	0.58–2.50	0.62			
Age (each year)	1.00	0.98–1.03	0.70			
Surgery (done versus not done)	0.42	0.14–1.24	0.11			
Radiotherapy (done versus not done)	0.50	0.23–1.10	0.09	0.10	0.03–0.32	***0.0003***
Chemotherapy (done versus not done)	0.73	0.33–2.29	0.79			
MGMT (each unit increase)	0.98	0.97–1.01	0.22	0.97	0.94–0.99	***0.0025***

MGMT (>19% versus ≤19%)	0.59	0.27–1.28	0.18	0.24	0.10–0.64	***0.0045***

95% CIs: 95% confidence intervals; HR: hazard ratio.

**Table 4:** Cox proportional hazard models predicting overall survival (dependent variable).

	Univariate analysis			Multivariable analysis		
	HR	95% CIs	*p* value	HR	95% CIs	*p* value
Gender (men versus women)	0.79	0.84–1.83	0.59			
Age (each year)	1.08	1.03–1.12	***0.0008***			
Surgery (done versus not done)	0.31	0.11–0.96	***0.0439***			
Radiotherapy (done versus not done)	0.08	0.02–0.24	***<0.0001***	0.10	0.02–0.42	***0.0021***
Chemotherapy (done versus not done)	0.26	0.07–1.01	0.052	0.15	0.03–0.86	***0.033***
MGMT methylation (each unit increase)	0.99	0.97–1.03	0.96			

MGMT methylation (>13% versus ≤13%)	0.57	0.24–1.31	0.19	0.33	0.12–0.92	***0.035***

95% CIs: 95% confidence intervals; HR: hazard ratio.
